# Chemical shielding of H_2_O and HF encapsulated inside a C_60_ cage

**DOI:** 10.1038/s42004-021-00569-0

**Published:** 2021-09-22

**Authors:** Samuel P. Jarvis, Hongqian Sang, Filipe Junqueira, Oliver Gordon, Jo E. A. Hodgkinson, Alex Saywell, Philipp Rahe, Salvatore Mamone, Simon Taylor, Adam Sweetman, Jeremy Leaf, David A. Duncan, Tien-Lin Lee, Pardeep K. Thakur, Gabriella Hoffman, Richard J. Whitby, Malcolm H. Levitt, Georg Held, Lev Kantorovich, Philip Moriarty, Robert G. Jones

**Affiliations:** 1 Physics Department, Lancaster University, Lancaster, UK; 2 Institute for Interdisciplinary Research, Jianghan University, Wuhan, China; 3 The School of Physics and Astronomy, The University of Nottingham, Nottingham, UK; 4 Fachbereich Physik, Universität Osnabrück, Osnabrück, Germany; 5 School of Physics and Astronomy, University of Leeds, Leeds, UK; 6 Diamond Light Source, Diamond House, Harwell Science & Innovation Campus, Didcot, Oxfordshire, UK; 7 School of Chemistry, The University of Southampton, Southampton, UK; 8 Department of Physics, King’s College London, London, UK; 9 School of Chemistry, The University of Nottingham, Nottingham, UK

**Keywords:** Carbon nanotubes and fullerenes, Molecular self-assembly, Scanning probe microscopy

## Abstract

Molecular surgery provides the opportunity to study relatively large molecules encapsulated within a fullerene cage. Here we determine the location of an H_2_O molecule isolated within an adsorbed buckminsterfullerene cage, and compare this to the intrafullerene position of HF. Using normal incidence X-ray standing wave (NIXSW) analysis, coupled with density functional theory and molecular dynamics simulations, we show that both H_2_O and HF are located at an off-centre position within the fullerene cage, caused by substantial intra-cage electrostatic fields generated by surface adsorption of the fullerene. The atomistic and electronic structure simulations also reveal significant internal rotational motion consistent with the NIXSW data. Despite this substantial intra-cage interaction, we find that neither HF or H_2_O contribute to the endofullerene frontier orbitals, confirming the chemical isolation of the encapsulated molecules. We also show that our experimental NIXSW measurements and theoretical data are best described by a mixed adsorption site model.

## Abstract (web summary)

The chemical nature of molecules encapsulated within fullerenes remain debated, with reports proposing a Faraday cage effect. Here, the authors show that H_2_O and HF molecules encapsulated inside a C_60_ cage experience a substantial intra-cage electrostatic interaction that results in off-center locations; despite this, the endofullerene’s frontier orbitals is unaffected, resulting in chemical shielding of the caged molecule.

## Introduction

The extent to which trapped, encapsulated, or otherwise confined atoms and molecules are influenced by changes in the external electrostatic environment is a fascinating problem. Endohedral fullerenes^1–3^ are an especially important molecular class from this perspective, with their properties exploited in areas as diverse as nanoelectronic components^4,5^, qubit candidates^6^, and ‘tracer’ species or contrast agents^7,8^. Of particular interest is the proposal that C_60_ acts as a Faraday cage^9–13^, screening out electrostatic fields from its interior, and allowing near-complete decoupling of the encapsulated species from the outside world. The extent of electrostatic decoupling remains, however, very much an open question.

‘Molecular surgery’^14^ has provided exciting new opportunities to study much larger fullerene-encapsulated species than previously possible. One of the most well-studied examples is H_2_O@C_60_^14–17^, in which the encapsulated water molecule is free of any hydrogen-bonded partner. Ortho-para nuclear spin conversion has been observed in H_2_O@C_60_ by nuclear magnetic resonance^18–20^, infra-red spectroscopy^16,18^, and pulsed terahertz spectroscopy^17^. Remarkably, ortho-para conversion has also been detected in this system by temperature-dependent capacitance measurements of bulk H_2_O@C_60_ crystals^21^, strongly suggesting that, despite the shielding offered by the surrounding fullerene cage, the encapsulated water molecule can have a significant influence on the external environment. HF@C_60_ has more recently been synthesized^22^ and provides a key comparative system to that of H_2_O@C_60_, not least because of the strong similarity in the dipole moments of free H_2_O and HF.

Hampered by the absence of clear experimental evidence, there has been considerable debate about the extent to which H_2_O interacts with the C_60_ cage^4,23–25^. Calculations of charge transfer result in a wide range of values^26^. Although some simulations suggest that the water molecule in H_2_O@C_60_ is electrostatically isolated^24^, other reports suggest that the position and orientation of the encapsulated H_2_O can be tuned via electric, magnetic, or photon excitation^4^, mechanical force^27^, or static (or dynamic) fields^28^.

**Fig. 1:**
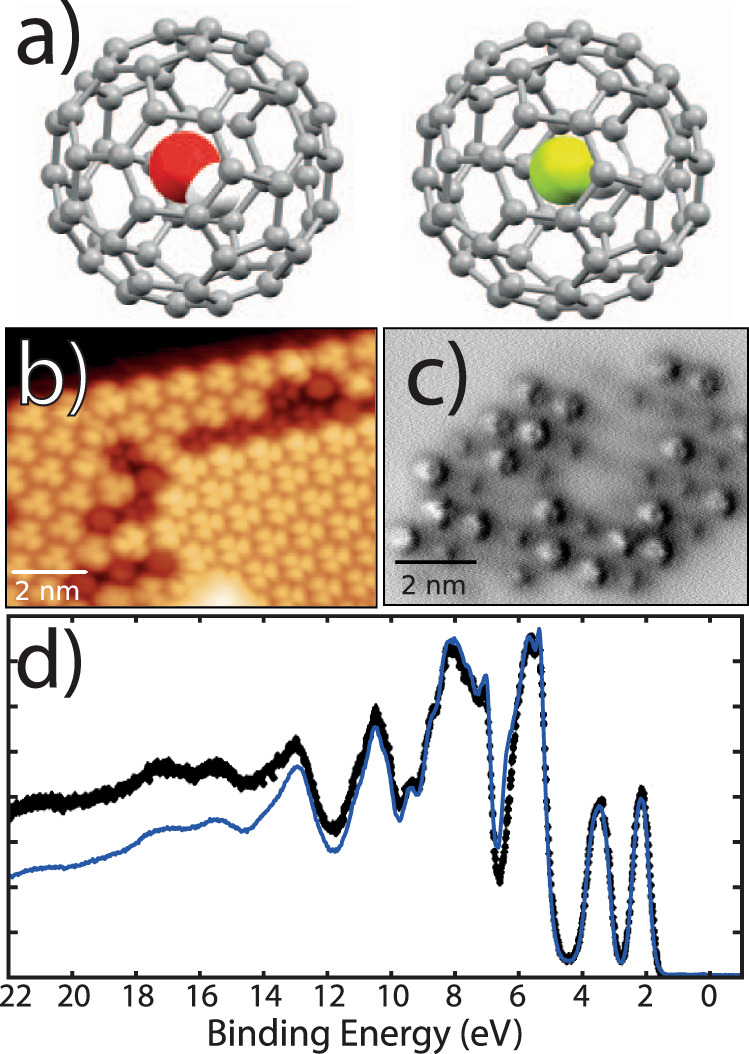
Endofullerene as a Faraday cage? **a** Ball-and-stick models of H_2_O@C_60_ and HF@C_60_. **b** STM image of a mixed (70 : 30) H_2_O@C_60_ : C_60_ monolayer island on Cu(111) deposited at room temperature. The bright and dark molecules have a height difference of 200 pm resulting from the reconstruction of the underlying Cu(111) surface. **c** Constant height ncAFM image of a different island atop a reconstructed Cu(111) surface exhibiting clear variation in molecular height. **d** Normalized valence band photoemission (synchrotron-based with *hν* = 110 eV) of a thick film of a 95% pure sample of H_2_O@C_60_ (blue line) shown with reference data for empty C_60_ (black line^35^). The scanning probe and photoemission data all point to a substantial screening of the encapsulated molecule. All SPM data were acquired at 5 K. Parameters: **b** 1.5 V/10 pA; **c** Oscillation amplitude, *a*_0_ = 300 pm, *V* = −2.1 mV.

Here we provide direct experimental measurement of the location of two separate encapsulated molecules, H_2_O and HF, each caged within buckminsterfullerene, C_60_, as illustrated in Fig. 1a. Through single-molecule scanning tunnelling microscopy, non-contact atomic force microscopy (ncAFM), and valence band photoemission, we show that the frontier orbital structure of the C_60_ molecule is unaffected by the presence of water inside the cage. Using the normal incidence X-ray standing wave (NIXSW) technique for the H_2_O@C_60_ : Ag(111) system, we determine the intra-cage location of both H_2_O and HF. The measured heights of the H_2_O and HF molecules above the Ag(111) surface are in good agreement with dispersion-corrected density functional theory (DFT) and molecular dynamics (MD) calculations, confirming the lack of chemical interaction of the encapsulated molecules with the surrounding carbon cage, yet revealing the strong influence of an adsorption-induced intra-fullerene electric field.

## Results

### C_60_ as a Faraday cage?

The local density of states of H_2_O@C_60_ was probed using scanning tunnelling microscopy (STM) measurements of a 70 : 30 mixture of empty and water-filled cages, deposited as a submonolayer on Cu(111). Analysis of C_60_ (sub)monolayers prepared at room temperature is complicated by the well-studied underlying surface reconstruction induced by fullerene adsorption^29^. Detailed low-energy electron diffraction (LEED) studies by a number of groups have provided compelling evidence that fullerene-induced vacancy reconstruction is prevalent on Cu(111) and Ag(111) surfaces^30–32^, with STM reports suggesting a mixed picture of ‘bright’ and ‘dark’ molecules^33,34^. To confirm the presence of surface reconstruction, we compared STM measurements with constant height ncAFM imaging (Fig. 1b, c), which clearly reveals that the mixture of ‘bright’ and ‘dark’ features in Fig. 1b is indeed due to the variation of the geometric height of the adsorbed molecules. This most likely arises from a combination of atom-top and vacancy adsorption sites; the brightness variations observed in the STM image of Fig. 1b are unrelated to water encapsulation (see Supplementary Information and Supplementary Fig. S1).

Despite STM’s exceptional sensitivity to minor changes in electronic structure, we found no difference between the appearance of empty and filled C_60_ molecules. This observation was confirmed across a wide range of different imaging parameters (Supplementary Fig. S1), strongly suggesting that the frontier orbitals of the fullerene are unperturbed by the presence of H_2_O. Similarly, ncAFM measurements, including force–distance analysis designed to compress individual fullerene cages, revealed no discernible differences (Supplementary Fig. S3) above the noise floor of our measurements. These observations were further confirmed using valence band X-ray photoemission spectroscopy (VB-XPS) at the Diamond Light Source (Beamline I09) collected at a photon energy of 110 eV. VB-XPS of a bulk film of 95% pure H_2_O@C_60_ (thick enough to completely attenuate the Ag 3*d* core-level photoelectron peaks from the substrate) was indistinguishable from that of C_60_ within the limits of the natural, and substantial, linewidth broadening. There is no discernable difference between the spectrum shown in Fig. 1d and that of empty C_60_, acquired at the same photon energy (see, in particular, ref. ^35^). The lack of an oxygen contribution to the highest occupied molecular orbital (HOMO) and HOMO + 1 features was also verified via resonant photoemission at the O K-edge (see Supplementary Information and Supplementary Fig. S2).

The fact that STM, ncAFM, and photoemission spectroscopy (including resonant photoemission) are each unable to distinguish between filled and empty C_60_ strongly suggests that neither electronic structure, the stiffness of the cage, nor the dielectric properties of the fullerene are appreciably affected by the presence of H_2_O (somewhat at odds with other findings^21^.) This seems to point towards a substantial screening of the encapsulate by the surrounding fullerene cage.

### Locating trapped molecules with NIXSW

**Fig. 2:**
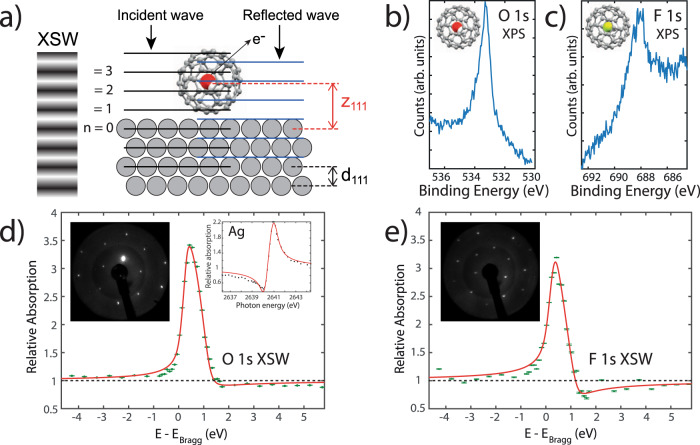
Locating trapped molecules with NIXSW. **a** Diagram of the NIXSW technique where an X-ray standing wave is established at the sample surface by tuning *hν* close to the crystal Bragg condition. **b** O 1*s* core-level photoemission spectrum (*hν* = 700eV) and **c** F 1*s* level (*hν* = 900 eV) for H_2_O@C_60_ and HF@C_60_, respectively, confirming the presence of the encapsulated molecules in the monolayer film. **d** NIXSW data for H_2_O@C_60_ with insets: bulk Ag NIXSW and low-energy electron diffraction (LEED, *E* = 46.5 eV). Fitting analysis results in a coherent position and coherent fraction of *P*_c_ = 0.35 ± 0.03 and *F*_c_ = 0.78 ± 0.05, respectively. **e** NIXSW data for HF@C_60_. Fitting analysis results in a coherent position and coherent fraction of *P*_c_ = 0.43 ± 0.03 and *F*_c_ = 0.72 ± 0.07, respectively. Inset, LEED (*E* = 50.5 eV). LEED confirms both samples are prepared as a single layer (2$\sqrt{3}$ × 2$\sqrt{3}$)R30° molecular superlattice on Ag(111). The NIXSW data were acquired at 20 K at the Ag{111} Bragg energy. *Y*-axis error bars in **d** and **e** are determined from fits of the O 1*s* and F 1*s* spectra used to determine relative absorption.

Given the inability of scanning probes and photoemission to distinguish between filled and empty fullerene cages, we turned to the NIXSW technique^36–38^, illustrated in Fig. 2a, to determine the intra-cage position of each encapsulated molecule. The location of H_2_O was probed via the O 1*s* core-level photoelectron peak excited at the Ag{111} Bragg energy, whereas HF was located using F 1*s* photoemission. Figure 2a, b show the O 1*s* and F 1*s* photoemission peaks acquired with a photon energy of 700 and 900 eV, respectively (the lower photon energies, as compared to that used for the NIXSW measurements (*hν* = 2637 eV at 20 K), were chosen to enhance the photoabsorption cross-section for high-resolution photoemission measurements).

Ag(111) was chosen as a substrate for these synchrotron-based measurements, in order to exploit the well-defined (2$\sqrt{3}$ × 2$\sqrt{3}$)R30° molecular superlattice that forms on the silver surface^30,39^. Results were collected at temperatures ranging from 20 K up to 200 K, with particular care taken to limit the degree of both extrinsic water adsorption at low temperatures and beam damage (see Supplementary Information).

NIXSW results collected at 20 K for H_2_O@C_60_ and HF@C_60_ are shown in Fig. 2d, e, respectively (see also Supplementary Figs. S4–S6 in the Supplementary Information file). The LEED patterns shown in the insets confirm the (2$\sqrt{3}$ × 2$\sqrt{3}$)R30° ordering of the molecular (sub)monolayer for both samples. The H_2_O@C_60_ XSW data averaged across the entire temperature range (see Supplementary Fig. S6) are best fit using a coherent position, *P*_c_, of 0.36 ± 0.01 and a coherent fraction, *F*_c_, of 0.72 ± 0.06 (see ref. ^36^ for a detailed explanation of the coherent position and coherent fraction parameters). These measurements translate to an oxygen atom position, *z*_111_(*O*), of 5.57 ± 0.03 Å above the Ag(111) surface. In comparison, and when again averaged across all temperatures, HF@C_60_ results in a coherent position, *P*_c_, of 0.40 ± 0.05 and a coherent fraction, *F*_c_, of 0.62 ± 0.07, placing the fluorine atom at a position, *z*_111_(*F*) = 5.7 ± 0.1 Å above the Ag(111) surface. The uncertainties in *z*_111_ in each case are an upper limit, determined from repeated measurements across different temperatures and sample preparations. Our NIXSW measurements show that the H_2_O and HF molecules are close to the centre of their respective fullerene enclosures.

### DFT and MD simulations

The results of the NIXSW measurements were compared to DFT calculations carried out within the Vienna ab initio simulation package (VASP)^40^ framework, using DFT-D3^41^ to account for dispersion forces. Calculations for a single C_60_ molecule were checked against a DFT-D3 simulation of the (2$\sqrt{3}$ × 2$\sqrt{3}$)R30° cell (Supplementary Fig. S10), where the adsorption heights were found to be almost identical. The remaining discussion therefore focusses on a single fullerene either adsorbed on an atom-top site or above a single-atom vacancy^30^, both with a hexagonal face of the C_60_ cage aligned parallel to the surface.

**Fig. 3:**
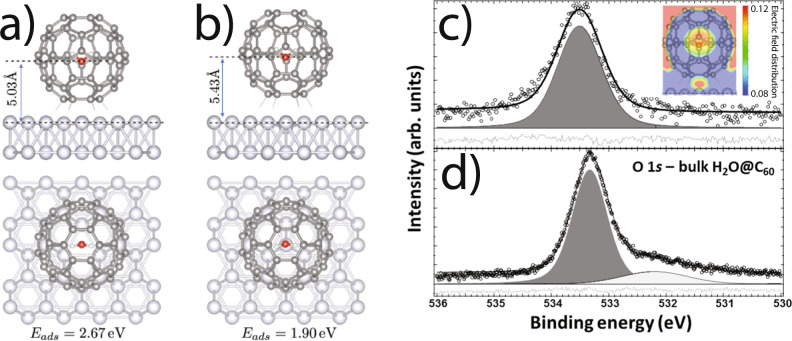
Dispersion-corrected DFT of H_2_O@C_60_. Ball-and-stick schematic showing DFT VASP results for H_2_O@C_60_ : Ag(111) in **a** vacancy and **b** atom-top sites. The oxygen-Ag(111) separation is 5.03 Å and 5.43 Å, respectively. **c**, **d** O 1s photoelectron spectra for the H_2_O@C_60_ monolayer and bulk film, respectively. Inset to **c** shows the DFT-predicted electric field distribution inside the cage. The broad additional peak in **d**, centred at ~532.2 eV, arises from contaminant material in the bulk film and is removed in the monolayer sample during the additional 650 K anneal.

Models of the DFT-D3-calculated geometries for H_2_O@C_60_ in the vacancy and atom-top arrangements are shown in Fig. 3a, b (see Supplementary Fig. S11 for HF@C_60_). A key observation is that, on adsorption, there are significant offsets in the position of the internal molecule relative to the centre of the C_60_ cage. For H_2_O@C_60_ and HF@C_60_ adsorbed in the vacancy site, the bottom layer of the C_60_ cage is calculated to be 1.89 Å above the surface Ag layer, resulting in a centre cage position of 5.17 Å. This is compared to an oxygen position of 5.03 Å and a fluorine position of 5.24 Å, i.e., the oxygen atom is located 0.14 Å closer to the surface, whereas for HF the fluorine is located 0.07 Å away from the surface. It is noteworthy that a detailed calculation of the potential energy surface of the frozen HF molecule inside the fixed C_60_ cage has also been reported^42^ using the DF-MP2 method^43^.

We find that the oxygen atom is located at 5.03 Å or 5.43 Å above the surface Ag(111) layer for the vacancy and atom-top structures, respectively—values that are both significantly different from that determined via the NIXSW measurements, i.e., 5.57 ± 0.03 Å. A similar discrepancy between experiment and DFT calculations is observed for the HF@C_60_ data: e.g., DFT calculations (VASP) for the relaxed vacancy model predict a separation between the fluorine atom and the uppermost Ag(111) layer of 5.24 Å, as compared to the NIXSW measurement of 5.7 ± 0.1 Å.

**Fig. 4:**
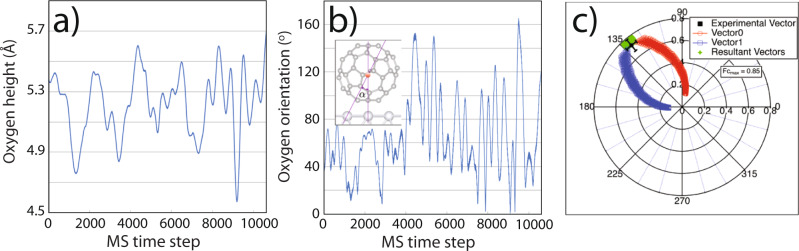
Dynamics and disorder in endohedral fullerenes. Molecular dynamics simulations (*T* = 180 K) showing **a** the height of the O atom of the intra-cage H_2_O molecule above the Ag(111) surface and **b** the angle between the H_2_O *C*_2_ axis and the vertical (as defined in the inset). **c** Average *F*_c_(*T*) (as radius length) and *P*_c_(*T*) (as angle) values for H_2_O@C_60_ plotted on an Argand diagram, and their decomposition into two component vectors labelled 0 and 1. Black error bars show uncertainty in *P*_c_ and *F*_c_ plotted as an experimental vector.

Calculated rotational energy barriers^44^ for water in H_2_O@C_60_ are of the order of 8 meV—the encapsulated molecule is extremely mobile. We similarly found that MD simulations (for *T* = 180 K, and within the CP2K-DFT-D3 framework^45^) revealed significant internal rotational motion. Figure 4a, b show plots of the internal molecular height and angle respectively, resulting in an average height increase of 0.3 Å relative to the ground state DFT calculation. This means that the average height is therefore 5.33 Å and 5.73 Å for the H_2_O molecule in the vacancy and atop structures, respectively (as discussed in the Supplementary Information, temperature-dependent NIXSW measurements showed little variation in the 20–200 K range. See Supplementary Fig. S6).

### Probing the intermolecular potential

The contribution of disorder notwithstanding, the theoretically predicted intra-cage positions of the encapsulated H_2_O and HF are due to charge redistribution from chemisorption of the C_60_ molecule on Ag(111)^46–48^, producing an intra-cage electric field with which the internal molecules interact (Fig. 3c inset). Good experimental evidence for the inhomogeneous electric field in H_2_O@C_60_ comes from O 1*s* photoelectron spectra. For a bulk endofullerene film, the O 1*s* peak associated with the encapsulated water has a Gaussian full width at half maximum (FWHM) of 0.7 eV, whereas for the chemisorbed monolayer the Gaussian width (under the same analyser operating conditions) increases almost 50% to 1.0 eV (compare Fig. 3c, d). The O 1*s* peak position, as referenced to the Ag 3*d* core level-binding energy, also shifts by ~200 meV. Given the lack of chemical interaction with the surrounding C_60_ cage, a plausible explanation for this 200 meV shift is that it arises from the difference in screening of the photogenerated core hole due to the modification of the intra-cage electrostatic environment.

Moreover, MD calculations semi-quantitatively explain the experimentally observed broadening of the O 1*s* photoemision peak and its shift to higher energies as compared to the bulk measurements. The O 1*s* core level shift (CLS) was approximately calculated^49^ as the energy required to excite the O 1*s* core electron for a sampling of different geometries of the H_2_O molecule inside the C_60_ cage obtained during the MD simulations (see Supplementary Information). We find that the FWHM value of the distribution of the O 1*s* CLSs for the adsorbed H_2_O@C_60_ is indeed larger than that for the gas-phase endofullerene, although the MD simulations produce a somewhat smaller increase in FWHM as compared to that seen in experiment (0.1 eV vs. 0.3 eV.) Similarly, the O 1*s* binding energy determined via this calculation shifts towards higher energy, again in agreement with experiment, although the magnitude of the shift is overestimated as compared to the experimental value (0.20 eV vs. 0.46 eV). Considering the approximate character of the method used for the calculation^49^, we would not expect full quantitative agreement. The fact that the calculation reproduces both the direction of the shift in binding energy and the increase in broadening of the core-level peak seen in the experiment provides good qualitative support for the presence and influence of the intra-cage electric field.

### Mixed adsorption sites

As noted above, C_60_ adsorption on metal (111) substrates is a surprisingly complex problem, being highly surface dependent^29^, and, as shown in Fig. 1c, is often associated with a significant inhomogeneity in molecular adsorption height. The majority of STM studies indicate a mixed picture^33,34^ of both ‘bright’ and ‘dark’ fullerenes (similar to those in Fig. 1b), where variations in brightness arise from a combination of atom-top and vacancy sites. In an influential piece of work, Li et al.^30^ suggested that C_60_ molecules on Ag(111) situate in vacancy sites, evidenced by a compelling combined LEED and DFT study. In a follow-up report^32^, the same authors extended this model and determined that the (2$\sqrt{3}$ × 2$\sqrt{3}$)R30° structure in fact comprised a mixture of C_60_ fullerenes on vacancy and atom-top sites. Indeed, our own LEED I(V) modelling (see Supplementary Fig. S9 and associated discussion) using the DFT coordinates from Fig. 3, suggests that a mixture of the two adsorption sites is also highly likely in our system, giving rise to an appreciable amount of static disorder in the positions of the encapsulated molecules with respect to the Ag(111) substrate and significantly complicating the XSW analysis.

To provide insight into whether the experimental NIXSW data are consistent with the mixed adsorption model, we explored the parameter space via an Argand diagram analysis. In this approach, the experimental values for *F*_c_(*T*) and *P*_c_(*T*) are plotted as a vector, as shown in Fig. 4c. We decompose the experimental vector into two components, which we label 0 and 1, assigned to the vacancy and atom-top adsorption sites, respectively. The vector combinations are then varied and recombined into a resultant (see Supplementary Information and Supplementary Figs. S7 and S8 for details), to represent different ratios of on-top and vacancy sites within the mixed film. A key difficulty in interpreting the NIXSW data in this way is that the underpinning equations are essentially under-determined and can therefore not be used to extract unique values of coherent fraction and coherent position. Despite this, the Argand analysis (summarized in Fig. 4c and detailed at length in the Supplementary Information file) illustrates that the NIXSW data are broadly consistent with a mixture of on-top and vacancy adsorption sites, suggesting that the measured oxygen position of *z*_111_(O), i.e., 5.57 ± 0.03 Å, can be interpreted as a combination of the two calculated positions of 5.33 Å and 5.73 Å for the H_2_O molecule in the vacancy and atop structures, respectively.

## Conclusions

In conclusion, our results address long-standing questions regarding the extent to which fullerene-encapsulated molecules, in particular H_2_O, are electrostatically screened and decoupled from their external environment. Although both H_2_O and HF contribute no discernable electronic state density to the frontier molecular orbitals of their surrounding C_60_—in other words, there is a distinct lack of orbital mixing and hybridization—adsorption on a metal surface (in this case, Ag(111)) causes a strong modification of the electrostatic potential within the cage. This in turn modifies the position of the encapsulated molecule as compared to that adopted in the gas-phase endofullerene^4^. Direct determination of the position of the intra-cage molecule is, however, very much complicated by the bonding geometry of the parent fullerene; a naive interpretation of the XSW data by itself fails to capture the many subtle contributions to the intra-cage energy balance and dynamics. Instead, the results of a comprehensive series of DFT, MD, and XSW analyses of the position of the encapsulated H_2_O and HF molecule can only be reconciled by taking into account both dynamic and static disorder in the position of the molecular encapsulate.

## Methods

### Scanning probe microscopy

Measurements were collected on a Createc GmbH LT STM-AFM system controlled by Nanonis electronics. Endohedral fullerenes were deposited via standard thermal sublimation under ultrahigh vacuum conditions (better than 1 × 10^−10^ mbar). For all scanning probe microscopy (SPM) measurements, we used a mixed monolayer film of C_60_ : H_2_O@C_60_ (in a 70 : 30 ratio) on a clean sputter-annealed Cu(111) surface. For reconstructed samples, the Cu(111) crystal was held at room temperature. A commercial qPlus sensor (Createc GmbH) with a separate tunnel current wire was used for both the STM and NC-AFM experiments (*f*_0_ ~ 20 kHz; *Q* ~ 30,000 at 5 K; nominal spring constant 1800 N m^−1^).

### Synchrotron XPS and NIXSW

Measurements were collected at beamline I09, Diamond Light Source. I09 is equipped with both a hard X-ray undulator, which was used for our XSW measurements at the Ag(111) Bragg energy of 2.63 keV, and a soft X-ray undulator, used for the acquisition of high-resolution C 1*s*, O 1*s*, F 1*s*, and valence band spectra. All synchrotron data were collected on an Ag(111) crystal prepared by standard sputter-annealing methods, after which 95% pure samples of H_2_O@C_60_ or HF@C_60_ were deposited via thermal sublimation. Considerable care was taken to reduce beam damage by detuning the beam and continuously moving across the crystal (see Section I.1 of the Supplementary Information for more detail.)

### DFT and MD calculations

Our relaxation calculations were based on DFT and were carried out using two ab initio codes: (i) the VASP^50^ that uses plane waves basis set and norm-conserving pseudopotentials, and (ii) the CP2K Quickstep package^45^, which employs hybrid Gaussian and plane-wave orbitals with a triple-zeta Gaussian molecularly optimized basis^51^, and Goedecker–Teter–Hutter pseudopotentials^52^. In both sets of calculations the Perde–Burke–Ernzerhof exchange-correlation density functional^53^ was used and the dispersion interactions were added via the Grimme DFT-D3 method^54^. The geometry optimization was considered complete when the forces on atoms were better than 0.01 eV Å. The Ag(111) surface was constructed using a slab model consisting of four atomic layers, where the atoms in the bottom two layers were kept fixed throughout all calculations. DFT simulations using VASP are regarded as being of higher precision due to the better approximation to a complete basis set that is possible with the inclusion of larger numbers of plane waves. As MD simulations were, however, only possible using CP2K due to high computational cost, we have also provided in Supplementary Fig. S11 CP2K results for two geometries of HF@C_60_ for validation purposes. MD simulations used a time step of 0.5 fs. Statistical analysis started from the 600th step, i.e., 0.3 ps after the NVT heat bath was applied. The temperature for MD was 180 K.

## Supplementary information

Supplementary Materials

## Data Availability

The experimental data and simulation files that support the findings of this study are available at 10.17635/lancaster/researchdata/483.

## References

[1] Lu X, Bao L, Akasaka T, Nagase S (2014). Recent progress in the chemistry of endohedral metallofullerenes. Chem. Comm..

[2] Lu X, Feng L, Akasaka T, Nagase S (2012). Current status and future developments of endohedral metallofullerenes. Chem. Soc. Rev..

[3] Rodriguez-Fortea A, Balch AL, Poblet JM (2011). Endohedral metallofullerenes: a unique host-guest association. Chem. Soc. Rev..

[4] Zhu C, Wang X (2015). Tuning the conductance of h2o@c-60 by position of the encapsulated h2o. Sci. Rep..

[5] Joachim C, Gimzewski J (1997). An electromechanical amplifier using a single molecule. Chem. Phys. Lett..

[6] Harneit, W. In *Endohedral Fullerenes: Electron Transfer and Spin* (ed. Popov, A.) 297–324 (Nanostructure Science and Technology, 2017).

[7] Bolskar RD (2008). Gadofullerene MRI contrast agents. Nanomedicine.

[8] Zhang J (2014). Gd3n@c-84(oh)(x): a new egg-shaped metallofullerene magnetic resonance imaging contrast agent. J. Am. Chem. Soc..

[9] Delaney P, Greer J (2004). C-60 as a Faraday cage. Appl. Phys. Lett..

[10] Chaur MN, Melin F, Ortiz AL, Echegoyen L (2009). Chemical, electrochemical, and structural properties of endohedral metallofullerenes. Ang. Chem. Int. Ed..

[11] Guha S, Nakamoto K (2005). Electronic structures and spectral properties of endohedral fullerenes. Coord. Chem. Rev..

[12] Shinohara H (2000). Endohedral metallofullerenes. Rep. Prog. Phys..

[13] Rubin, Y. In *Fullerenes and Related Structures. Topics of Current Chemistry*. Vol. 199, 67–91 (Springer, 1999).

[14] Kurotobi K, Murata Y (2011). A single molecule of water encapsulated in fullerene c-60. Science.

[15] Krachmalnicoff A, Levitt MH, Whitby RJ (2014). An optimised scalable synthesis of h2o@c60 and a new synthesis of h2@c60. Chem. Commun..

[16] Shugai, A. et al. Infrared spectroscopy of endohedral water in c_60_. Preprint at https://arxiv.org/abs/2102.06389 (2021). 10.1063/5.0047350 33810704

[17] Zhukov, S. S. et al. Rotational coherence of encapsulated ortho and para water in fullerene-c60 revealed by time-domain terahertz spectroscopy. *Sci. Rep.* **10**, 18329 (2020). 10.1038/s41598-020-74972-3 PMC7592058 33110105

[18] Beduz C (2012). Quantum rotation of ortho and para-water encapsulated in a fullerene cage. Proc. Natl Acad. Sci. USA.

[19] Mamone S (2014). Nuclear spin conversion of water inside fullerene cages detected by low-temperature nuclear magnetic resonance. J. Chem. Phys..

[20] Meier B (2018). Spin-isomer conversion of water at room temperature and quantum-rotor-induced nuclear polarization in the water-endofullerene h_2_O*@*c_60_. Phys. Rev. Lett..

[21] Meier, B. et al. Electrical detection of ortho-para conversion in fullerene-encapsulated water. *Nat. Commun*. **6**, 8112 (2015). 10.1038/ncomms9112 PMC4560827 26299447

[22] Krachmalnicoff A (2016). The dipolar endofullerene hf@c60. Nat. Chem..

[23] Dunn JL, Rashed E (2018). Evidence for Jahn-Teller effects in endohedral fullerenes. J. Phys. Conf. Ser..

[24] Kaneko S, Hashikawa Y, Fujii S, Murata Y, Kiguchi M (2017). Single molecular junction study on H2O@C60:H2O is “electrostatically isolated”. Chem. Phys. Chem..

[25] Zhu, C. & Wang, X. Transport properties of the h2o@c-60-dimer-based junction. *J. Phys. Condens. Matt*er **27**, 375301 (2015). 10.1088/0953-8984/27/37/375301 26325223

[26] Varadwaj, A. & Varadwaj, P. R. Can a single molecule of water be completely isolated within the subnano-space inside the fullerene C_60_ cage? A quantum chemical prospective. *Chem. Eur. J*. **18**, 15345 –15360 (2012). 10.1002/chem.201200969 23090782

[27] Min, K., Farimani, A. B. & Aluru, N. R. Mechanical behavior of water filled c60. *Appl. Phys. Lett*. **103**, 263112 (2013).

[28] Xu, B. & Chen, X. Electrical-driven transport of endohedral fullerene encapsulating a single water molecule. *Phys. Rev. Lett*. **110**, 156103 (2013). 10.1103/PhysRevLett.110.156103 25167287

[29] Ledieu J, Gaudry É, Fournée V, Smerdon JA, Diehl RD (2017). Fullerene adsorption on intermetallic compounds of increasing structural complexity. Z. Kristallogr. Crystalline Mater..

[30] Li, H. I. et al. Surface geometry of c-60 on ag(111). *Phys. Rev. Lett*. **103**, 056101 (2009). 10.1103/PhysRevLett.103.056101 19792515

[31] Pai, W. W. et al. Optimal electron doping of a c-60 monolayer on cu(111) via interface reconstruction. *Phys. Rev. Lett*. **104**, 036103 (2010). 10.1103/PhysRevLett.104.036103 20366662

[32] Pussi K (2012). Elucidating the dynamical equilibrium of c_60_ molecules on ag(111). Phys. Rev. B.

[33] Altman EI, Colton RJ (1993). Determination of the orientation of c_60_ adsorbed on au(111) and ag(111). Phys. Rev. B.

[34] Gardener JA, Briggs GAD, Castell MR (2009). Scanning tunneling microscopy studies of c_60_ monolayers on au(111). Phys. Rev. B.

[35] Brühwiler P, Maxwell A, Nilsson A, Martensson N, Gunnarsson O (1993). Auger and photoelectron study of the Hubbard U in C-60, K3C60, and K6C60.. Phys. Rev. B.

[36] Woodruff D (2005). Surface structure determination using x-ray standing waves. Rep. Prog. Phys..

[37] Jones R (2002). X-ray standing waves at surfaces. J. Phys. Condens. Matter.

[38] Zegenhagen J (1993). Surface-structure determination with x-ray standing waves. Surf. Sci. Rep..

[39] Pussi, K. et al. Elucidating the dynamical equilibrium of c-60 molecules on Ag(111). *Phys, Rev. B* **86**, 205406 (2012).

[40] Hafner, J. Ab-initio simulations of materials using VASP: density-functional theory and beyond. *J. Comp. Chem*. **29**, 2044–2078 (2008). 10.1002/jcc.21057 18623101

[41] Grimme, S., Antony, J., Ehrlich, S. & Krieg, H. A consistent and accurate ab initio parametrization of density functional dispersion correction (DFT-D) for the 94 elements H-Pu. *J. Chem. Phys*. **132**, 154104 (2010). 10.1063/1.3382344 20423165

[42] Kalugina YN, Roy P-N (2017). Potential energy and dipole moment surfaces for hf@c60: Prediction of spectral and electric response properties. J. Chem. Phys..

[43] Werner H-J, Manby FR, Knowles PJ (2003). Fast linear scaling second-order møller-plesset perturbation theory (mp2) using local and density fitting approximations. J. Chem. Phys..

[44] Farimani AB, Wu Y, Aluru NR (2013). Rotational motion of a single water molecule in a buckyball. Phys. Chem. Chem. Phys..

[45] Hutter J, Iannuzzi M, Schiffmann F, VandeVondele J (2014). Cp2k: atomistic simulations of condensed matter systems. Wiley Interdisc. Rev. Comp. Mol. Sci..

[46] Tjeng L (1997). Development of the electronic structure in a k-doped c-60 monolayer on a ag(111) surface. Sol. St. Comm..

[47] Yang W (2003). Band structure and Fermi surface of electron-doped c-60 monolayers. Science.

[48] Gibson AJ, Temperton RH, Handrup K, O’Shea JN (2017). Resonant core spectroscopies of the charge transfer interactions between c-60 and the surfaces of au(111), ag(111), cu(111) and pt(111). Surf. Sci..

[49] Köhler L, Kresse G (2004). Density functional study of CO on Rh(111). Phys. Rev. B.

[50] Kresse G, Furthmüller J (1996). Efficient iterative schemes for ab initio total-energy calculations using a plane-wave basis set. Phys. Rev. B.

[51] VandeVondele J, Hutter J (2007). Gaussian basis sets for accurate calculations on molecular systems in gas and condensed phases. J. Chem. Phys..

[52] Hartwigsen C, Goedecker S, Hutter J (1998). Relativistic separable dual-space gaussian pseudopotentials from h to rn. Phys. Rev. B.

[53] Perdew JP, Burke K, Ernzerhof M (1996). Generalized gradient approximation made simple. Phys. Rev. Lett..

[54] Grimme S, Antony J, Ehrlich S, Krieg H (2010). A consistent and accurate ab initio parametrization of density functional dispersion correction (dft-d) for the 94 elements h-pu. J. Chem. Phys..

