# Supplementary material for: Chemical shielding of H2O and HF encapsulated inside a C60 cage
Source: Commun Chem. 2021 Sep 22;4:135. doi: 10.1038/s42004-021-00569-0 (PMC9814403; doi:10.1038/s42004-021-00569-0)
Supplement: Supplementary file 1 — Supplementary Materials [file 42004_2021_569_MOESM1_ESM.pdf]

Chemical shielding of H_2O and HF encapsulated inside a C_{60} cage Supplementary Information

Samuel P Jarvis[1],* Hongqian Sang[2,8], Filipe Junqueira[3], Oliver Gordon[3], Jo E. Hodgkinson[3], Alex Saywell[3], Philipp Rahe[4], Salvatore Mamone[3], Simon Taylor[3], Adam Sweetman[5], Jeremy Leaf[3], David Duncan[6], Tien-Lin Lee[6], Pardeep K. Thakur[6], Gabriella Hoffman[7], Richard Whitby[7], Malcolm Levitt[7], Georg Held[6], Lev Kantorovich[8], Philip Moriarty[3],† and Robert G Jones[9]

[1] *Physics Department, Lancaster University, Lancaster, LA1 4YB, UK*

[2] *Institute for Interdisciplinary Research, Jianghan University, Wuhan 430056, China*

[3] *The School of Physics and Astronomy, The University of Nottingham, Nottingham NG7 2RD, UK*

[4] *Fachbereich Physik, Universität Osnabrück, Barbarastrasse 7, 49076 Osnabrck, Germany*

[5] *School of Physics and Astronomy, University of Leeds, Leeds, LS2 9JT, UK*

[6] *Diamond Light Source, Diamond House, Harwell Science & Innovation Campus, Didcot, Oxfordshire, OX11 0DE, UK*

[7] *School of Chemistry, The University of Southampton, Southampton, SO17 1BJ, UK*

[8] *Department of Physics, King's College London, The Strand, London, WC2R 2LS, UK and*

[9] *School of Chemistry, The University of Nottingham, Nottingham NG7 2RD, UK*

* samuel.jarvis@lancaster.ac.uk
† philip.moriarty@nottingham.ac.uk

I. EXPERIMENTAL MEASUREMENTS

Supporting measurements and analyses to which we refer in the main paper are described in the following sections.

I.1. Sample purity and limiting beam damage. We have interpreted the O 1s core-level spectra shown in Fig. 2 of the main paper as indicative of the presence of the H_2O molecule inside the fullerene cage. However, water is, of course, often a key residual contaminant in ultrahigh vacuum systems. We therefore took particular care during the measurements to monitor and minimise the possible contribution of adventitious water to the O 1s spectra. During sample preparation the Ag(111) surface was repeatedly sputter-annealed until no carbon or oxygen could be detected. Additionally, we note that all fullerene (sub)monolayers investigated in this study were prepared using identical sample preparation methods. In the case of HF@C_{60} this allowed us to check for an oxygen contamination signal, of which none could be found for a $(2\sqrt{3} \times 2\sqrt{3})$R30° molecular superlattice prepared under the same conditions as those for the H_2O@C_{60} sample. Furthermore, the intensity ratio of the C 1*s* and O 1*s* photoemission peaks was broadly in line with that expected for a single H_2O molecule inside a C_{60} cage, taking into account photoionisation cross-section and escape depth considerations.

Exposure of H_2O@C_{60} to full beam conditions was, however, found to cause considerable damage to the encapsulated water, noticeable by the appearance of an additional broad oxygen peak in XPS spectra. To limit this effect in our XSW measurements we used a variety of methods. This included reducing the x-ray intensity by detuning the undulator producing the incoming x-ray beam, conducting measurements at reduced sample temperatures, limiting the count time for measurements, and continuously moving the x-ray spot across the sample crystal between XSW sweeps. The effectiveness of this approach was checked by collecting O 1s XPS spectra immediately before and after every XSW measurement such that no noticeable change in the O 1s signal was apparent.

Extrinsic water adsorption at low temperatures was continuously monitored using regular O 1s XPS spectra between XSW measurements. Adsorption was minimised to negligible levels by time-limiting the experiments and, if required, the sample was warmed above 180 K to desorb any adventitious water.

I.2. Do the H_2O and fullerene orbitals mix in $H_2O@C_{60}$? As discussed in the main paper, we found no evidence for the intracage presence of water in STM and ncAFM measurements of mixed coverages of $H_2O@C_{60}$ and C_{60}. In figure S1 we show a series of STM measurements using a range of bias voltages and sample preparations. Samples investigated include thick multilayer films (Figure S1a) and monolayer films prepared at room temperature (Figure S1c-h). Additionally, in order to better compare filled and unfilled fullerenes without the complication of underlying surface reconstruction, in one series of experiments we suppressed the C_{60}-induced reconstruction of Cu(111) by preparing samples at 77 K, followed by a short anneal to $\sim$ 200 K, producing small molecular islands shown in Figure S1b).

Valence band photoemission (Fig. 1(d) of the main paper) also showed no evidence of the presence of a contribution of the molecular orbitals of H_2O to the fullerene density of states. However, given the large difference in the relative abundance of carbon and oxygen (a ratio of sixty carbon atoms to every oxygen atom), the total density of states is, of course, weighted very heavily towards the fullerene cage orbital contribution. We therefore used resonant photoemission (RESPES) at the oxygen K edge in an attempt to "amplify" any contribution of the encapsulated water to the frontier molecular orbitals of $H_2O@C_{60}$. As shown in Figure S2 below, however, the RESPES signal shows no enhancement arising from an oxygen-related contribution.

I3. Molecular compression as a test of cage occupancy? We also attempted to use force-distance measurements in ncAFM to distinguish between filled and empty fullerenes, i.e. to determine whether the compressibility of $H_2O@C_{60}$ was different to that of C_{60}. Representative data are shown in Fig.S3. Despite repeated attempts, we could not detect reproducible systematic differences in frequency shift-vs-z curves across a large number of molecules.

I.4. Representative NIXSW and reflectivity data; selecting best fit parameters. Figures S4 and S5 are data acquired at 20K showing the variation in the intensity of the O 1s and F 1s core-level photoemission peaks, respectively, as a function of photon energy in the NIXSW geometry. The figures also include the associated reflectivity curve and a contour map of the coherent position vs coherent fraction fitting "landscape". Furthermore, we observed very little change in the coherent fraction and coherent position as a function of temperature, as shown in Fig.S6 below, and therefore an average of the values measured for all temperatures was used to determine the position of the

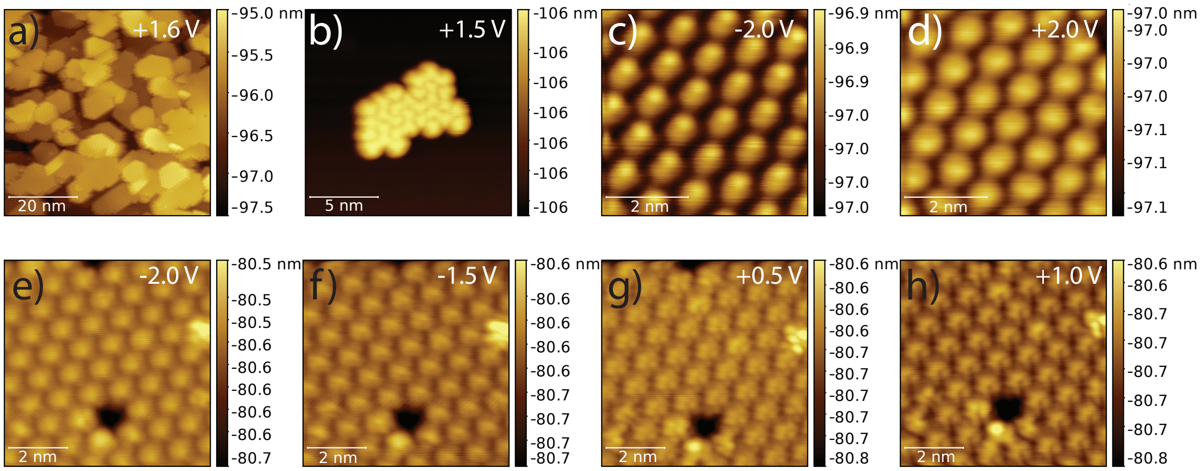

FIG. S1. Representative constant current STM images of mixed films of $H_2O@C_{60}$ and C_{60}. (a) Large scale image of a multilayer sample collected at +1.6V. (b) Small molecular island prepared by depositing molecules on a cooled (77 K) substrate. (c,d) Images collected at -2V and +2V respectively on a monolayer sample prepared at room temperature. (e-h) STM image sequence on another island collected at varying bias voltages.

encapsulated molecule in each case.

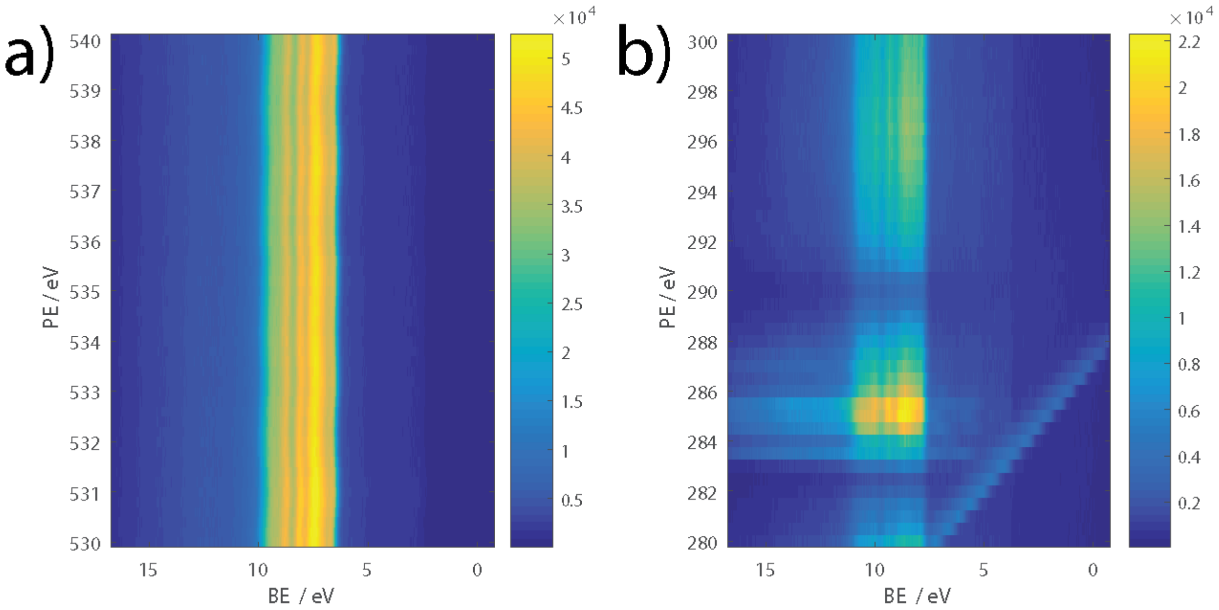

FIG. S2. 2D intensity plot of resonant valence band photoemission spectra recorded at the (a) oxygen K edge, and (b) carbon K edge. The spectra are plotted as a function of binding energy (horizontal axis) and photon energy (vertical axis). Although the C K edge map shows clear variation in intensity, the oxygen map shows no change when sweeping the K edge. Note that the feature running diagonally in the lower right corner of (b) arises from second-order excitation of the C 1s core-level.

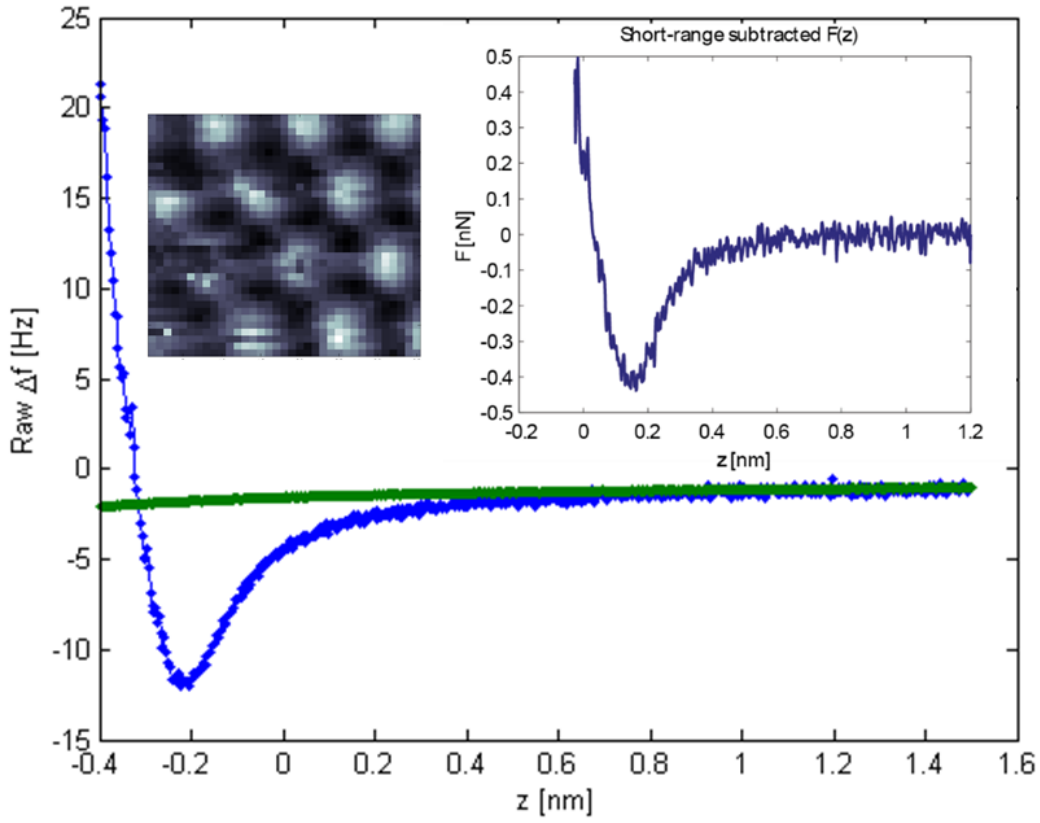

FIG. S3. Blue line: A typical frequency shift, Δf, vs tip-sample separation, z, acquired above an adsorbed fullerene on Cu(111) with a fullerene-terminated tip. Green line: Background curve acquired above a fullerene-free region of the Cu(111) surface. The insets show: **left:** a representative 2D slice though a 3D grid of $\mathrm{d}f(z)$ data, where each pixel represents a complete $\mathrm{d}f(z)$ curve; **right:** a short-range force-distance curve computed by subtracting the curves shown in the main figure and then applying the Sader-Jarvis algorithm[1] so as to invert the frequency shift measurements to force.

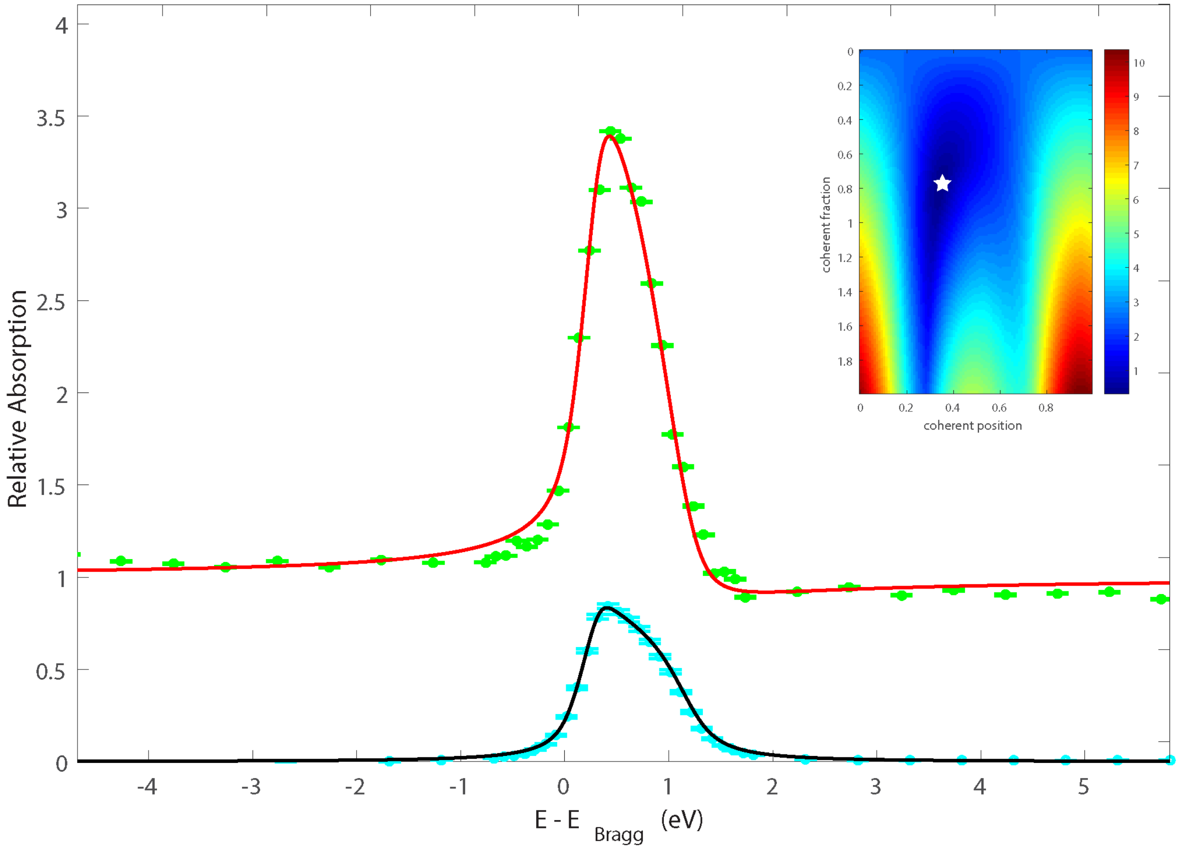

FIG. S4. XSW data (circles) and best fit (solid line) for H_2O@C60 derived from the O 1s photoemission peak, collected at 20 K, and shown with the associated reflectivity curve. Inset shows a contour map of the sum of squared residuals from the fit versus coherent fraction and coherent position for the XSW data, with the white star indicating the best fit region.

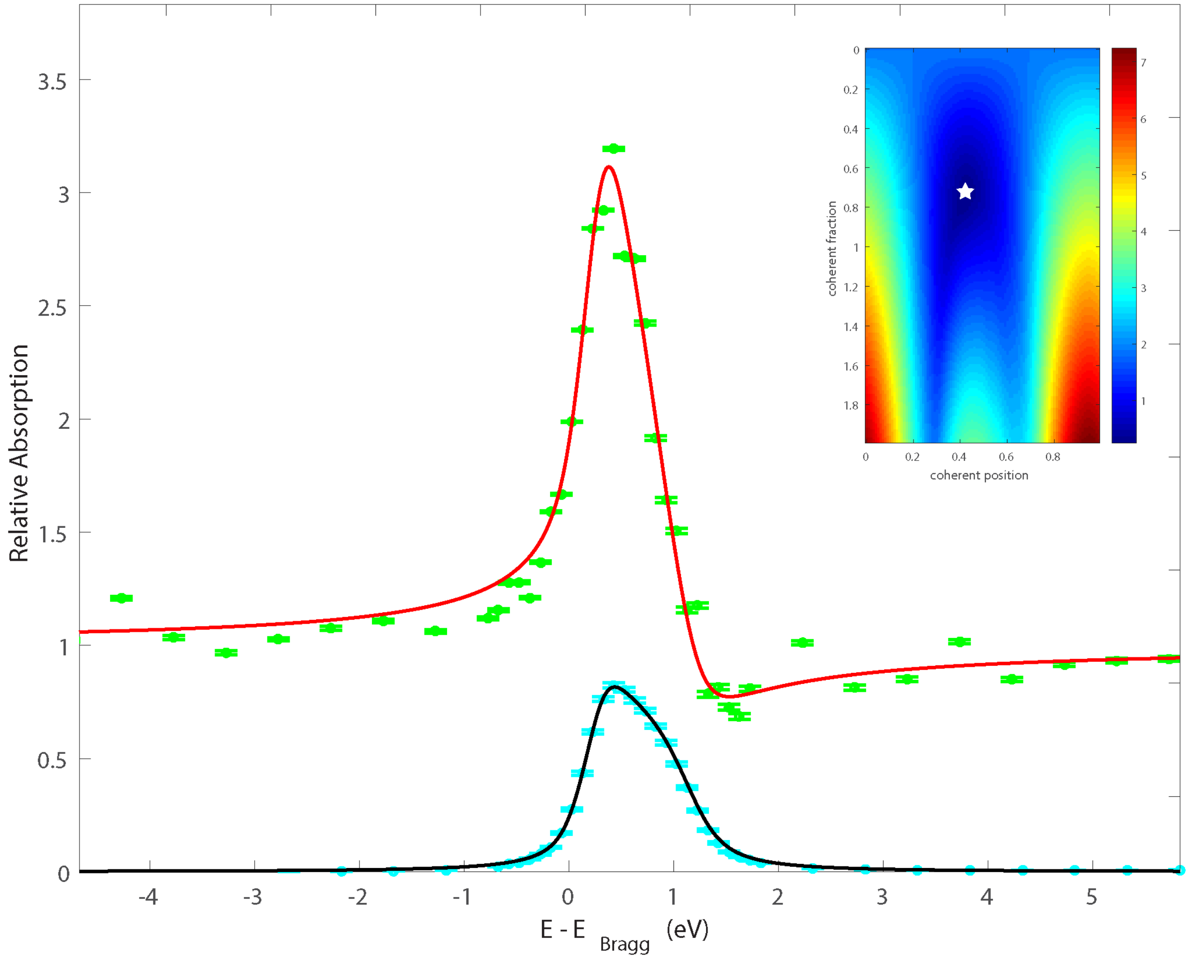

FIG. S5. XSW data (circles) and best fit (solid line) for HF@C60 derived from the F 1s photoemission peak, collected at 20 K, and shown with the associated reflectivity curve. Inset shows a contour map of the sum of squared residuals from the fit versus coherent fraction and coherent position for the XSW data, with the white star indicating the best fit region.

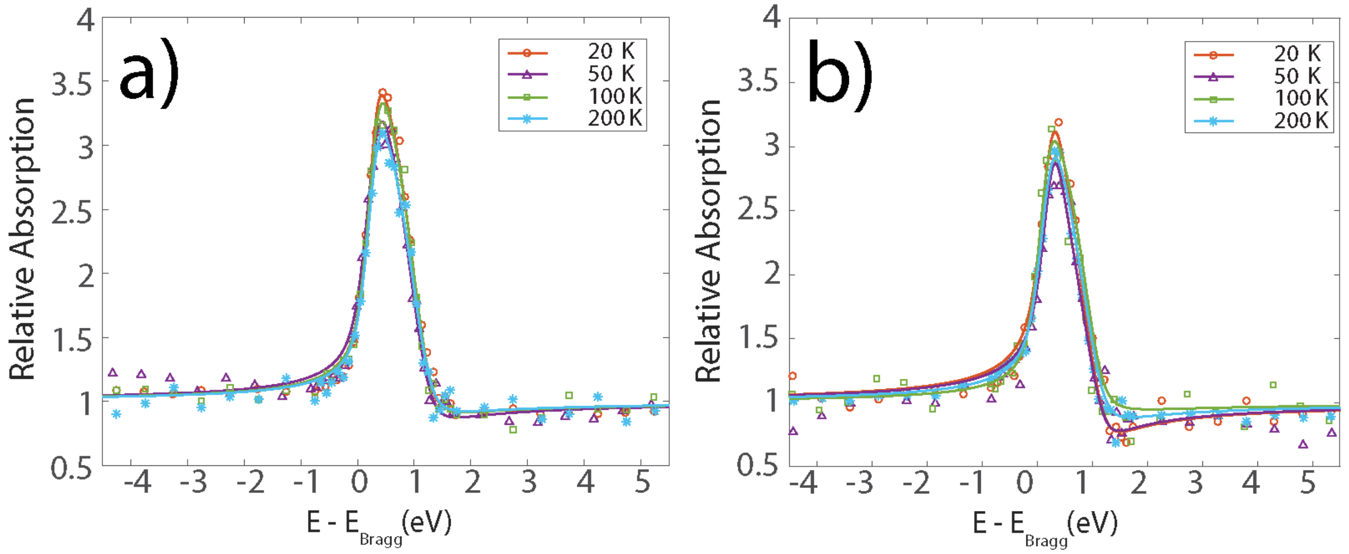

FIG. S6. **(a)** and **(b)**: overlaid NIXSW data (and corresponding fits) within a temperature range of 20 K - 300 K for $H_2O@C_{60}$ and $HF@C_{60}$ respectively.

I.5. NIXSW Argand diagram analysis

A. Two-site adsorption of $H_2O@C_{60}$ on Ag(111). Density functional theory calculations (see main paper and Section II below) suggest two possible adsorption sites for C_{60} (a lower vacancy site, and a higher atop-site) with a height difference of Δ z_{111} = 0.4 Å along the surface normal [111] direction. We assume that H_2O and C_{60} move as a unit relative to the (111) planes.

The Ag(111) layer spacing is D_{111} = 2.361 Å. The height difference of 0.4 Å≡ 0.169 (fractional = Δ z_{111}/D_{111}) ≡ 60.99° on the Argand diagram. Hence the two vectors corresponding to the oxygen atoms in the two adsorption sites are 61° apart. No systematic change in coherent position (P_c) or coherent fraction (F_c) of oxygen was observed for changes in temperature. The average of the four different temperature vectors is $P_c = 0.360$ (fractional) ≡ 0.850 Å≡ 129.6°and $F_c = 0.724$ (see Figure S S7). It is this vector that will be decomposed into two component vectors. Note that the oxygen atom within the C_{60} resides $2D_{111} + P_c$ above the surface (111) plane.

The standard deviation of the experimental coherent positions, P_c, for the four temperatures is 4.5° while the estimated error bars from the original fits are somewhat larger at ± 11°. The standard deviation of the experimental coherent fractions, F_c, for the four temperatures is 0.063 while the estimated error bars from original fits are also 0.063. We shall use errors of ±4.5°and ±0.063.

We define the outer position as site 1, represented by vector1 (length F_{c_1}, angle P_{c_1}) in the Argand diagram), and the inner position as site 0, vector0 (length F_{c_0}, angle P_{c_0} in the Argand diagram) , where the relative population, P1, of site 1 is given by F_{c_1} /(F_{c_0}+F_{c_1}). For a given substrate crystal there is a maximum possible coherent fraction, $F_{c_{max}}$ which depends on the perfection of the crystal and experimental parameters. If all the population adsorbs into into just two sites, $F_{c_{max}} = F_{c_0} + F_{c_1}$. If part of the adsorbate (with an absolute population of P_{rand}), adsorbs across all possible positions with respect to the surface normal then its contribution to the resultant vector is zero. In this case an effective maximum coherent fraction can be defined as $F_{c_{eff}}$ = $F_{c_{max}}$(1-P_{rand})= F_{c_0}+F_{c_1} for the ordered part of the surface. Rearrangement gives $F_{c_{max}} = F_{c_{eff}}/(1 - P_{rand})$.

A program has been written to decompose the average experimental vector into two component vectors, 0 and 1, which are 61° apart on the Argand diagram. In the program the component vectors at angles α°and α +61°are rotated from 0 to 360°while both their radii are systematically varied from 0 to 1. At each stage the two vectors are added to produce the resultant. Any resultant that takes a value within the uncertainty range of the experimental vector, and also with a given value of $F_{c_{max}}$ ±0.01, are extracted. A quality of fit was determined for each resultant, defined as the

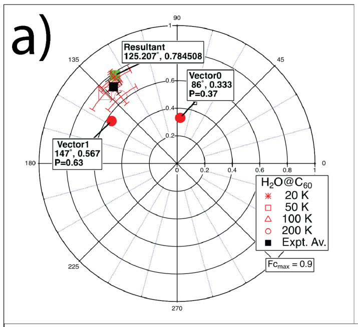

b)

Sites	Components	Cp/ °	Cp(frac)	P	Z_{111}/Å*	
Vacancy	Vector0	80	0.222	0.79	5.25	less probable
Atop	Vector1	141	0.391	0.21	5.65	
Vacancy	Vector0	99	0.275	0.5	5.37	most probable
Atop	Vector1	160	0.444	0.5	5.77	
Vacancy	Vector0	118	0.328	0.21	5.50	less probable
Atop	Vector1	179	0.497	0.79	5.90	

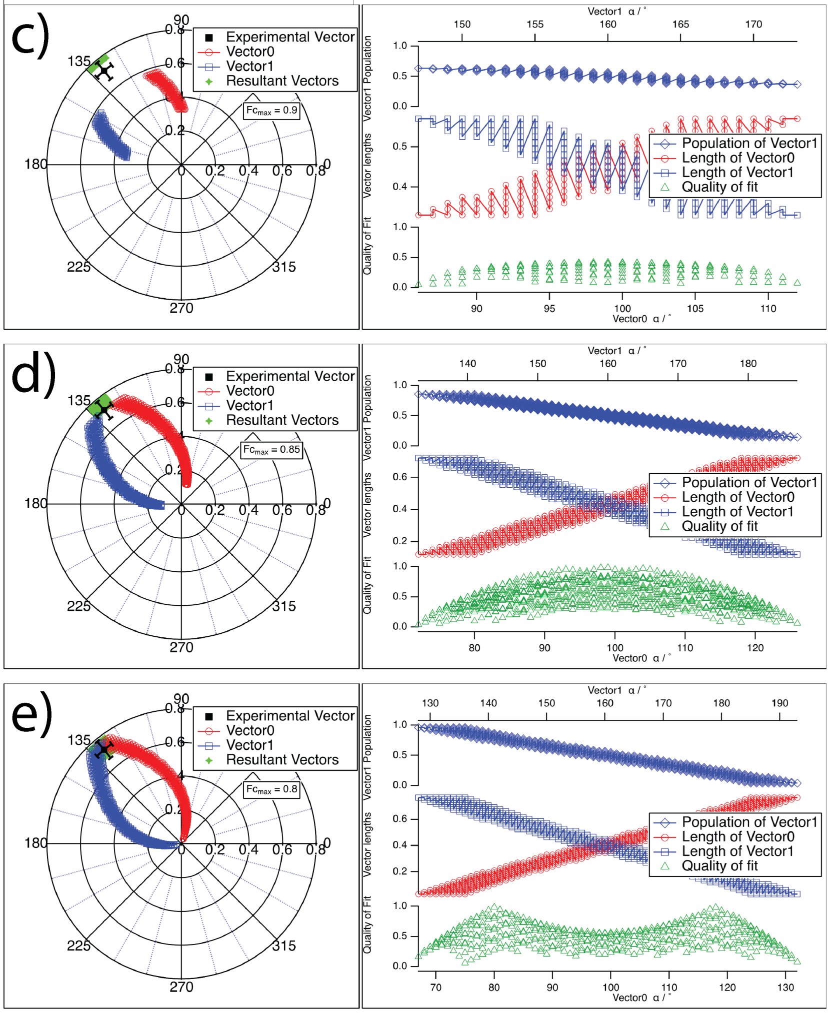

FIG. S7. Argand diagram analysis for NIXSW data on $H_2O@C_{60}$. (a) Argand diagram showing the four vectors representing the O/Ag(111) NIXSW data at four temperatures, 20, 50, 100 and 200 K, together with their average. Also shown are an example pair of component vectors at 86°& 147°with populations of 0.63 and 0.33. $F_{c_{max}} = 0.9$ was used in the calculation. (b) Table summarising the most likely vectors from (c-e). Vector pairs calculated for (c) $F_{c_{max}}$=0.9±0.01, (d) $F_{c_{max}}$=0.85±0.01 (reproduced in Figure 4 of the main paper), and (e) $F_{c_{max}}$=0.80±0.01, which give resultant vectors within the error bars of the experimental average. Left, Argand diagram showing the experimental average vector, with error bars; Vectors 0 and 1, and the resultant vectors lying within the error bar box. Right shows the angles and lengths of vector pairs, the corresponding population of vector1, and the quality of fit.

normalised distance from the error box edge to the resultant in the direction of the experimental value (= 0 outside and at the error box edge, increasing to 1 at the experimental value).

For $F_{c_{max}} = 0.92$, no solution exists. For $F_{c_{max}} = 0.9$, see Fig S7(c), a range of solutions for vectors1 and 2 exist with resultants that lie in the top part of the experimental error box. These solutions are also shown in FigS7(c), as the vector pairs angles and lengths, the corresponding population of vector1, and the quality of fit for a given pair. The pairs of P_c and F_c values range from (86°, 0.33) & (147°, 0.57), through to (112°, 0.57) & (173°, 0.33) with the range of values of P_c ± 4.5°and F_c ± 0.05. The first of these vector pairs is shown in Fig S S7(a) as an example. The quality of fit parameter shows that the best fits are in the middle of the angular range at (99°, 0.45) & (160°, 0.45) where the populations of both vectors are equal at 0.5, with the quality of fit always less than 0.42. If the absolute population of random distributed oxygen were $P_{rand} = 0.05$, then the true $F_{c_{max}}$ = 0.947.

For $F_{c_{max}}$= 0.85, see Fig. S7(d) and Figure 4 of the main paper, solutions exist with resultants filling the top half of the error box. The pairs of P_c and F_c values range from (72°, 0.12) & (133°, 0.72) through to (126°, 0.72) & (187°, 0.12). The quality of fit parameter maximises at (99°, 0.42) & (160°, 0.42) at a value of 0.98 where the relative populations of vectors 0 and 1 both equal 0.5. As the populations become unequal, the quality of fit drops. $F_{c_{max}}$ =0.85 here would correspond to a true value of $F_{c_{max}} = 0.90$ or 0.94 if $P_{rand} = 0.05$ or 0.1 respectively.

For $F_{c_{max}}$ =0.80, see Fig. S7(e), solutions exist with resultants filling the whole of the error box. The pairs of P_c and F_c values range from (67°, 0.03) & (128°, 0.765) to (132°, 0.765) & (193°, 0.03) which include solutions of a single component vector at 129.6°with the other vector having zero intensity. The quality of fit parameter maximises in two places for (80°, 0.165) & (141°, 0.63) where vector1 has a population of 0.79, and (118°, 0.63) & (179°, 0.165) with vector1 population=0.21. For equal populations the quality of fit is still a substantial 0.55. $F_{c_{max}}$ =0.80 here would correspond to $F_{c_{max}}$ = 0.84, 0.90, 0.94 if P_{rand}=0.05, 0.1, 0.15 respectively.

This can be summarised as a model having P_{rand}=0.05 and a 0.5:0.5 relative populations of two sites with oxygen located at Z_{111} = 5.37±0.13Å(Vacancy site) and 5.77±0.13Å(Atop site). The errors correspond to changes in occupation to 0.2:0.8 for positive error and 0.8:0.2 for negative error.

B. Two-site adsorption of HF@C$_{60}$ on Ag(111). This analysis follows the same procedure as the H$_2$O@C$_{60}$ analysis above. The two vectors corresponding to the two fluorine positions are 61° apart.No systematic change in coherent position (P_c) or coherent fraction (F_c) was observed for changes in temperature. The average of the four different temperature vectors is $P_c = 0.407$ (fractional) ≡ 0.961 Å≡ 146.5°and $F_c = 0.623$. The standard deviation of the experimental P_c values for the four temperatures is 16.96°while the estimated error bars from the original fits are ± 15°. The standard deviation of the experimental coherent fractions, F_c, for the four temperatures is 0.066 while the estimated error bars from original fits are ± 0.08. We have used errors of ±17°and ± 0.066.

For $F_{c_{max}} \geq 0.81$, no solution exists. For $F_{c_{max}} = 0.80 \pm 0.01$, see Fig.S8(c), a range of solutions for vectors0 and 1 exist with resultants that lie in the top part of the experimental error box. The pairs of P_c and F_c values range from (94°, 0.33) & (155°, 0.465), through to (138°, 0.465) & (199°, 0.33) with the range of values of P_c ± 15°and F_c ± 0.08. The first of these is shown in Fig.S8(a) as an example. The quality of fit parameter are all rather low with a maximum of 0.33 in the middle of the angular range at (115.5°, 0.40) & (176.5°, 0.40) where the populations of both vectors are equal at 0.5. The $F_{c_{max}} = 0.8$ value also corresponds to higher values of 0.84, 0.89, 0.94 if $P_{rand} = 0.05$, 0.1, 0.15 respectively.

For $F_{c_{max}} = 0.75$, see Fig.S8(d), solutions exist with resultants filling the top half of the error box. The pairs of P_c and F_c values range from (80°, 0.15) & (141°, 0.60) through to (152°, 0.60) & (213°, 0.15). The quality of fit parameter maximises at 0.75 for vectors (116°, 0.375) & (177°, 0.375) where the relative populations of both equal 0.5. As the populations become unequal, the quality of fit drops zero at the extremes. $F_{c_{max}}$ =0.75 here would correspond to $F_{c_{max}}$ = 0.79, 0.83, 0.88 if P_{rand} = 0.05, 0.1, 0.15 respectively.

For $F_{c_{max}}$ =0.70, see Fig.S8(e), solutions exist with resultants filling the whole of the error box. The pairs of P_c and F_c values range from (70°, 0.015) & (131°, 0.675) to (162°, 0.675) & (223°, 0.015) which include solutions of a single component vector at 146.5°(the experimental average) with the other vector having zero intensity. The quality of fit parameter maximises in two places for (101°, 0.195) & (162°, 0.51) where vector1 has a population of 0.72, and (131°, 0.51) & (192°, 0.195) with vector1 population=0.28. For equal populations the quality of fit is still a substantial 0.69. $F_{c_{max}}$ = 0.70 here would correspond to $F_{c_{max}}$ = 0.74, 0.78, 0.82 if N_{rand} = 0.05, 0.1, 0.15 respectively of the adsorbate. A full filling of the error bars is only obtained for $F_{c_{eff}}$ = 0.7, which corresponds to a true value of $F_{c_{max}}$ = 0.82 if 15 % of the surface (N_{rand}=0.15) is randomly distributed. This is a

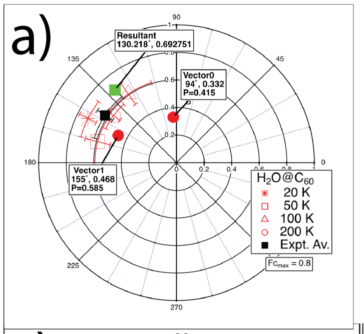

b)

Sites	Components	Cp/°	Cp(frac)	P	Z_{111}/Å *	
Vacancy	Vector0	83	0.23	0.87	5.27	less
Atop	Vector1	144	0.40	0.13	5.67	probable
Vacancy	Vector0	116	0.32	0.5	5.48	most
Atop	Vector1	177	0.49	0.5	5.88	probable
Vacancy	Vector0	149	0.41	0.87	5.69	less
Atop	Vector1	210	0.58	0.13	6.09	probable

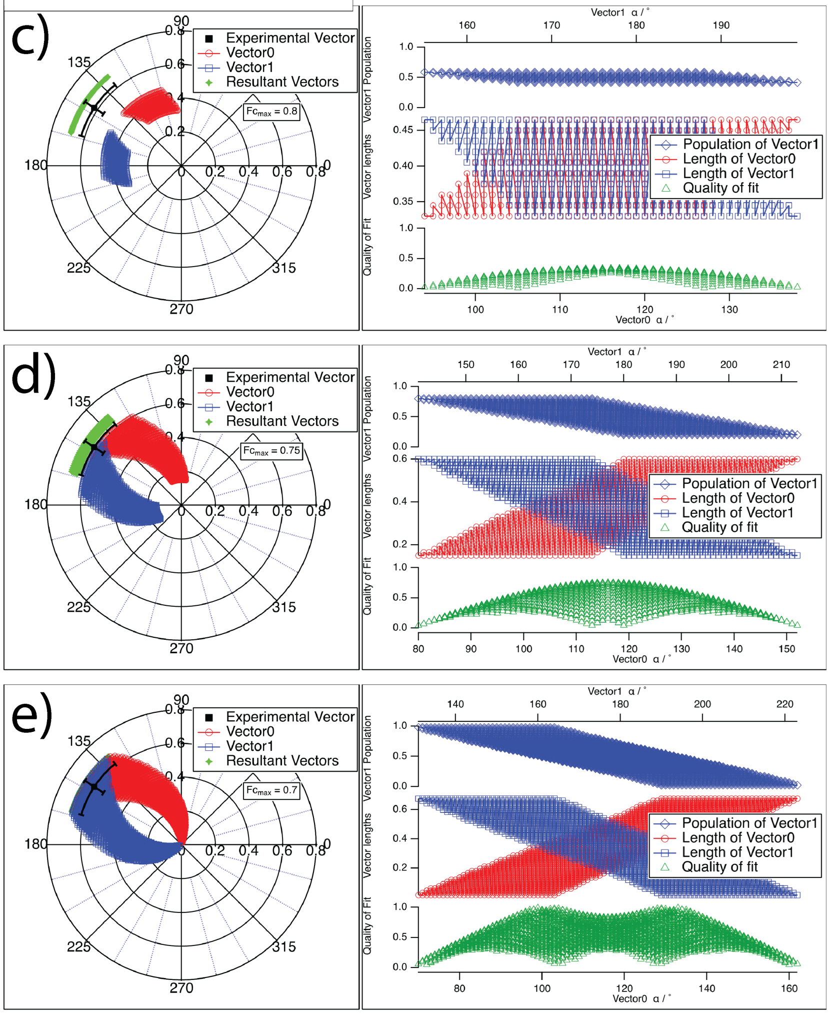

FIG. S8. Argand diagram analysis for NIXSW data on HF@C_{60}. (a) Argand diagram showing the four vectors representing the F/Ag(111) NIXSW data at four temperatures, 20, 50, 100 and 200 K, together with their average. Also shown are an example pair of component vectors at 94°& 155°with populations of 0.59 and 0.42. $F_{c_{max}} = 0.8$ was used in the calculation. (b) Table summarising the most likely vectors from (c-e). Vector pairs calculated for (c) $F_{c_{max}}$=0.8±0.01, (d) $F_{c_{max}}$=0.75±0.01 (reproduced in Figure 4 of the main paper), and (e) $F_{c_{max}}$=0.70±0.01, which give resultant vectors within the error bars of the experimental average. Left, Argand diagram showing the experimental average vector, with error bars, Vectors0 and 1, and the resultant vectors lying within the error bar box. Right shows the angles and lengths of vector pairs, the corresponding population of vector1, and the quality of fit.

reasonable value for $F_{c_{max}}$.

The table in Fig.S8(b) shows a summary of the analysis where the quality of fit ≥ 0.5 has been used as a guide and where a true $F_{c_{max}}$ is expected to lie between 0.85 and 0.95. This can be summarised as a model having $P_{rand} \sim 0.10$ and a 0.5:0.5 relative population of two sites with fluorines located at $Z_{111} = 5.48 \pm 0.21$Å(vacancy site) and 5.88 ± 0.21Å(atop site). The errors correspond to changes in population to 0.13:0.87 for positive error and 0.87:0.13 for negative error.

I7. LEED I-V calculations. LEED intensity-vs-energy (I-V) curves were calculated using the "CLEED" program package [2, 3]. This program package employs fully dynamical scattering theory along the lines of algorithms developed by Pendry [4] and Van Hove / Tong [5]. Bulk scattering was calculated by Pendry's layer doubling method with bulk inter-layer spacings of 2.35 Å for Ag{111}. Convergence is typically achieved by including 8 bulk layers. The scattering phase shifts for all atoms within the structure were calculated as a function of energy using the program package provided by Barbieri and Van Hove [6]. The maximum angular momentum quantum number used in the calculations, l_{max}, was 9, the radial root mean square (rms) displacement was 0.03 Å for Ag and 0.08 for C atoms in accordance with earlier work [7, 8].

I-V curves were calculated for three different $p(2\sqrt{3} \times 2\sqrt{3})R30°$ geometries: (rec) with an Ag vacancy below each C_60 molecule and vertical buckling of 0.09 Å of the remaining 11 Ag atoms in the top-most layer; (unrec) with no Ag vacancy and a maximum buckling of 0.32 Å in the first layer of Ag atoms; (flat) no buckling in the first and second Ag layer. The (rec) and (unrec) geometries were extracted from the DFT results. The (flat) geometry used the average vertical coordinates of the first and second Ag layers, respectively, in the (unrec) geometry.

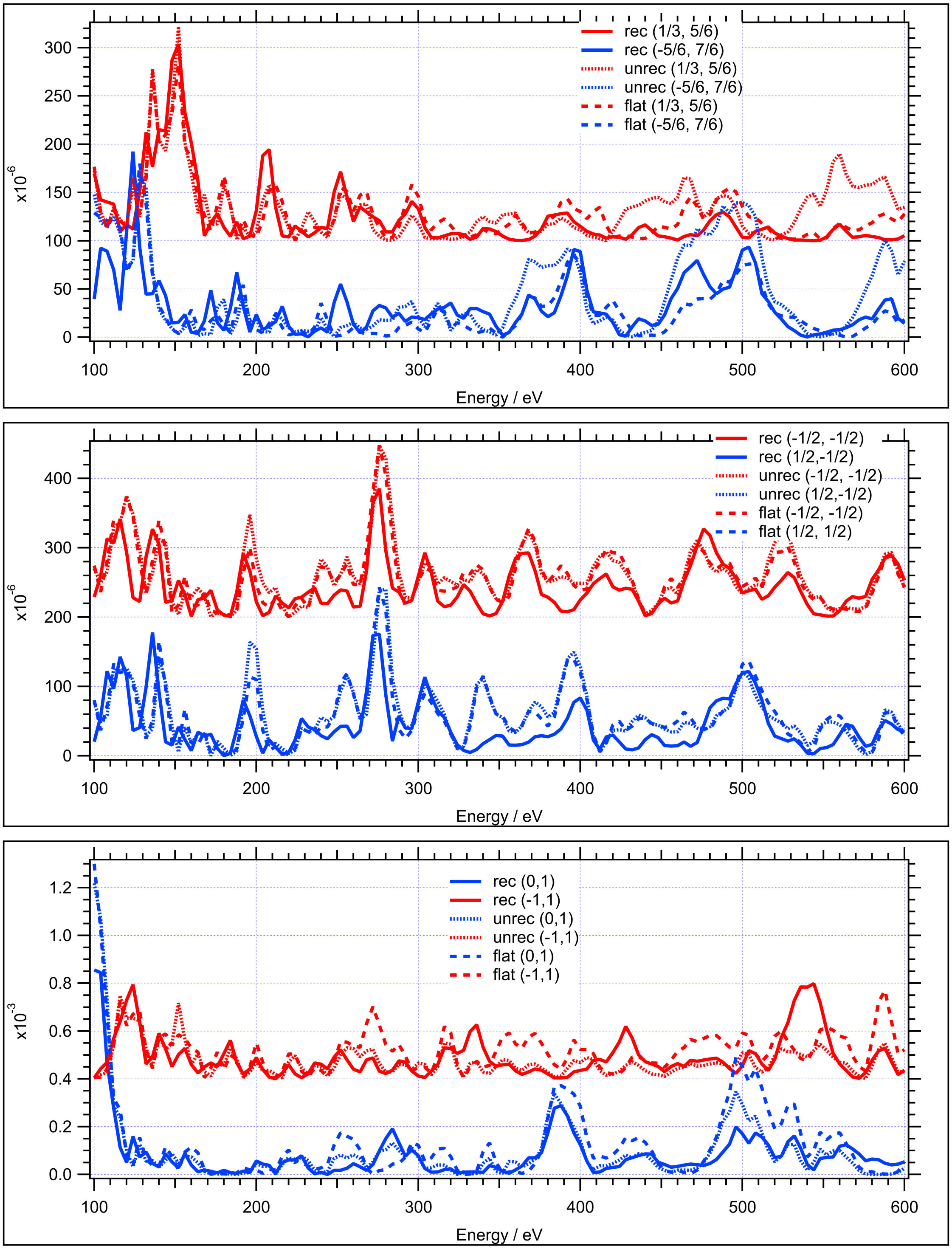

FIG. S9. LEED I(V) curves for fractional and integer order diffraction spots for three different geometries: **rec**, where there is a Ag vacancy below each C_{60} molecule and vertical buckling of the uppermost layer; **unrec**, with no Ag vacancy but with buckling; and **flat**, with no buckling.

SUPPLEMENTARY NOTE: THEORY AND SIMULATIONS

Modelling the endofullerene adsorption site. In Fig.S10 we show the results of a DFT-D3 calculation with four $H_2O@C_{60}$ molecules arranged in vacancy sites on a $(2\sqrt{3} \times 2\sqrt{3})R30°$ primitive cell, calculated using the VASP code. The adsorption heights were found to change very little compared to the single molecule calculations, resulting in an increased oxygen height of 5.06 Å compared to 5.04 Å for the single molecule structure. Fig.S11 shows ball-and-stick geometries for the two local minima configurations calculated for $HF@C_{60}$ in the vacancy surface site using both the VASP (a and b) and CP2K (c and d) codes. The two configurations correspond to either the F atom 'pointing' up (a,c) or down (b,d) with respect to the surface. Of the two, the 'down' configuration has the lowest energy configuration (by 74 meV) resulting in a F height of 5.24 Å with respect to the Ag(111) surface layer as calculated with VASP.

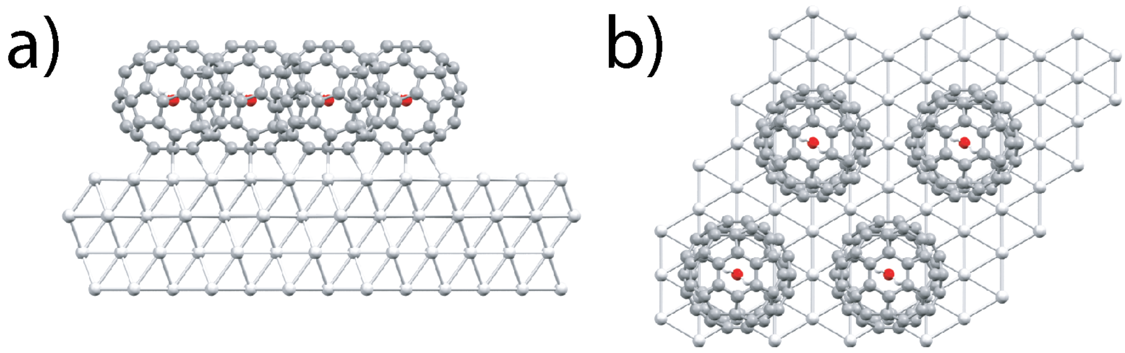

FIG. S10. Ball-and-stick schematic showing relaxed coordinates from a DFT-D3 calculation with four $H_2O@C_{60}$ molecules arranged in vacancy sites on a $2(\sqrt{3}$ x $2\sqrt{3})R30°$ primitive cell. The C_{60} fullerene adsorption height was found to be almost identical to the geometry consisting of only a single fullerene. This similarity appears to imply that whilst intermolecular interactions are key to overcoming the barriers involved with reconstruction, once achieved, the C_{60}-surface interaction becomes dominant.

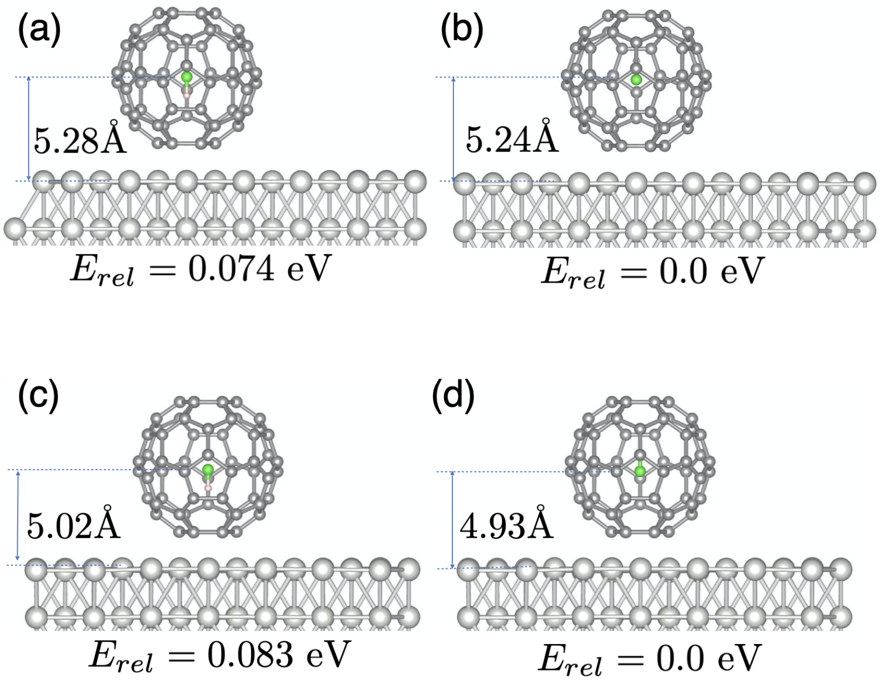

FIG. S11. Relaxed geometry of HF@C_{60} as calcuated by dispersion-corrected DFT. Ball-and-stick schematic showing VASP DFT-D3 results for HF@C_{60}:Ag(111) in vacancy sites in (a) "F-up" and (b) "F-down" orientations of HF. Ball-and-stick schematic showing CP2K DFT-D3 results for HF@C_{60}:Ag(111) in vacancy sites in (c) "F-up" and (d) "F-down" orientations of HF.

[1] Sader, JE; Jarvis, SP. *Appl. Phys. Lett.*, **84**, 1801 (2004)

[2] Held, G.; Wander, A.; King, D. A. *Physical Review B (Condensed Matter)* **1995**, *51*, 17856–17866

[3] G. Held and W. Braun; CLEED manual. available from the authors

[4] Pendry, J. B. *Low Energy Electron Diffraction*; Academic Press: London, 1974

[5] Van Hove, M. A.; Tong, S. Y. *Surface Crystallography by LEED*; Springer Series in Solid–State Sciences; Springer: Berlin, 1979

[6] Barbieri, A.; Van Hove, M. A. Phase shift program package. available from http://electron.lbl.gov/software/software.html

[7] W. Braun and G. Held *Surf. Sci.* **2005**, *594*, 203–211

[8] Zhasmina V. Zheleva and Tugce Eralp and Georg Held *J. Phys. Chem. C* **2012**, *116*, 618-625
